# Osteogenic Marker Expression after SHED and PMMA–HA Scaffold Application in Alveolar Bone Defects

**DOI:** 10.1055/s-0045-1812311

**Published:** 2025-11-17

**Authors:** Tania Saskianti, Renata Damayanti, Fami W. Pangestika, Ardianti M. Dewi, Rini D. Ridwan, Choon Y. Fan, Masami Kanawa, Takeshi Kawamoto, Kotaro Tanimoto, Katsumi Fujimoto

**Affiliations:** 1 Department of Pediatric Dentistry, Faculty of Dental Medicine, Universitas Airlangga, Surabaya, Indonesia; 2 Biomaterial and Tissue Engineering Research Center, Universitas Airlangga, Surabaya, Indonesia; 3 Department of Oral Biology, Faculty of Dental Medicine, Universitas Airlangga, Surabaya, Indonesia; 4 Department of Oral & Maxillofacial Surgical Science, Faculty of Dentistry, MAHSA University, Petaling Jaya, Malaysia; 5 Department of Biomedical Science, Research and Development Division, Natural Science Center for Basic Research and Development, Hiroshima University, Hiroshima, Japan; 6 Writing Center, Hiroshima University, Hiroshima, Japan; 7 Department of Orthodontics, Graduate School of Biomedical and Health Sciences, Hiroshima University, Hiroshima, Japan; 8 Department of Molecular Biology and Biochemistry, Graduate School of Biomedical and Health Sciences, Hiroshima University, Hiroshima, Japan

**Keywords:** biomedical, polymethylmethacrylate, hydroxyapatite, scaffold, SHED, alveolar bone defect therapy

## Abstract

**Objective:**

Alveolar bone defects in dentistry—caused by trauma, congenital anomalies, and periodontal disease—can substantially impair oral health and tooth function. These effects pose significant challenges in dentistry, and current gold-standard treatments such as autogenous bone grafting have notable limitations. Stem cell from human exfoliated deciduous teeth (SHED) has great potential for bone tissue engineering. The aim of this study was to evaluate the effect of SHED seeded in polymethylmethacrylate (PMMA)-hydroxyapatite (HA) scaffolds on the expression of osteogenic markers in alveolar bone defects.

**Materials and Methods:**

A total of 24 male Wistar rats had their maxillary incisors extracted, and the resulting defects were randomly assigned to three groups: (1) no treatment (control), (2) defects treated with PMMA–HA, and (3) defects treated with SHED seeded in PMMA–HA scaffolds. Immunoreactivity of BMP2, RUNX2, ALP, TGF-β, OCN, OPG, RANK, and RANKL was assessed.

**Results:**

Immunohistochemical results showed upregulated expression of BMP2, RUNX2, ALP, TGF-β, OCN, and OPG and downregulated expression of RANK and RANKL in rats treated with SHED-seeded PMMA–HA scaffolds compared with control and defects treated with PMMA–HA only groups.

**Conclusion:**

SHED seeded into PMMA–HA scaffolds effectively influenced the expression of several osteogenic markers, such as BMP2, RUNX2, ALP, TGF-β, OCN, OPG, RANK, and RANKL, in alveolar bone defects. These findings indicate that the combination of SHED and PMMA–HA scaffolds could potentially become a promising regenerative alternative to autogenous bone grafting in alveolar bone defects.

## Introduction

Alveolar bone defects are a common occurrence in dentistry, often resulting from various conditions such as tooth extractions, periodontal disease, trauma, cleft palate, tumors, odontogenic cysts, dehiscence, and fenestrations. 1 The prevalence of alveolar bone defects caused by dentoalveolar trauma, according to the World Health Organization (2023), in children aged up to 12 years is 20% in 1 billion people, affecting 11 to 30% of primary teeth and 5 to 20% of permanent teeth. These data indicate that there is still much work to be done, considering that alveolar bone defects left untreated can lead to resorption of the alveolar bone. 2 Therefore, alveolar bone grafting is needed to repair these defects.

Up to now, autogenous bone has been considered the gold standard for bone grafts due to its osteogenic, osteoconductive, and osteoinductive properties that support osteogenesis. 3 4 However, the limited availability of autogenous bone, along with its associated postoperative complications, has hindered its use. 3 5 To address this problem, regenerative dentistry offers a promising contemporary approach to alveolar bone defect repair through tissue engineering. The scaffold, stem cells, and growth factors—known as the triad of tissue engineering—play a crucial role in this process. 3

Stem cells from human exfoliated deciduous teeth (SHEDs) are a type of mesenchymal stem cell (MSC) derived from the oral cavity that can regenerate damaged tissue, including repairing alveolar bone defects and restoring function. SHEDs show higher proliferation than dental pulp stem cells and bone marrow-derived stem cells. 6 Moreover, SHED can also differentiate into various cell types, such as neuro-like cells, osteogenic, adipogenic, and chondrogenic cells. 7 Previous studies have shown SHED's ability to enhance bone formation and wound healing in vivo. 8

Polymethyl methacrylate (PMMA), which has been used in dentistry as an orthodontic retainer, obturator, temporary crown, and denture material, 8 9 is now being more frequently utilized as a scaffold in tissue engineering because of its low toxicity, biocompatibility, excellent mechanical stability, ease of manipulation, and cost-effectiveness. 9 10 11 However, PMMA has some disadvantages, including poor osteointegration and limited bone attachment, which need to be enhanced biologically. Furthermore, this polymer also lacks adhesive properties to bone surfaces and exhibits no bioactivity.

Several studies have found that PMMA, when combined with hydroxyapatite (HA), shows improved mechanical and biological characteristics. 12 HA is a ceramic material that can be found in nature or synthetically produced. It exists naturally in the mineral form of calcium apatite, with the formula Ca _10_ (PO _4_ ) _6_ (OH) _2_ , and is widely used as a bone graft material due to its excellent osteoconductive and osteointegrative properties. Nonetheless, HA needs to be combined with other materials, as it is brittle, has low tensile strength, and poor plasticity, making it difficult to manipulate. 13 14

Additionally, the combination of PMMA and HA has been shown to increase the mechanical strength of the biomaterial. 15 A combinatory ratio of PMMA:HA at 20:80 has been considered optimal for synthetic graft materials (see Supplementary Materials , available in the online version only). This combination also exhibits traits important for a bone graft material candidate, such as antibacterial and antifungal properties and nontoxic effects on SHED and osteoblasts. 2 16

Although various studies have been conducted on biomaterials, research on the use of SHED seeded in PMMA–HA scaffolds for bone regeneration in alveolar bone defects remains limited. To optimally facilitate the application of SHED–PMMA–HA as a biomaterial candidate for alveolar bone defect repair, it is important to understand the underlying molecular mechanisms responsible for its osteogenic potential. BMP2, RUNX2, ALP, TGF-β, OCN, OPG, RANK, and RANKL are crucial osteogenic markers involved in bone remodeling. BMP and TGF-β play substantial roles in osteoblast differentiation, leading to the expression of RUNX2, OCN, and ALP. 17 18 19 OPG, synthesized by osteoblasts, plays an important role in osteoclastogenesis and promotes bone formation by blocking the interaction between RANKL and RANK. 18 19

This study hypothesizes an upregulation in the expression of BMP2, RUNX2, ALP, TGF-β, OCN, and OPG and downregulation in the expression of RANK and RANKL during alveolar bone defect repair using SHED-seeded PMMA–HA scaffolds. Therefore, the objective of this study is to evaluate the effect of SHED seeded in PMMA–HA scaffolds on the expression of osteogenic markers in alveolar bone defects.

## Materials and Methods

### Ethical Clearance

All procedures involving animals were approved by the Institutional Animal Ethics Committee of the Faculty of Dental Medicine, Universitas Airlangga, Surabaya, Indonesia (No. 239/HRECC.FODM/V/2021), and were carried out in compliance with the principles of the 3Rs ( *r* eplacement, *r* eduction, and *r* efinement).

### Study Design and Animal Experimental Preparation

This is a post-test-only control group study in which subjects were randomly assigned to experimental and control groups, and outcome measures were assessed exclusively following the intervention. The sample size was calculated using the formula: *n* = {2 *σ* ^2^ ( *Z*_ 1- *α*_ + *Z*_ 1- *β*_ )2}/( *µ* 1 − *µ* 2)2, where *σ* = 2.01, *Z*_1_ – *α* = 1.96, and *Z*_1_ – *β* = 1.28 (based on standard normal distribution values). Substitution of these parameters yielded a sample size of approximately 2.98, which was rounded up to 3 animals per group. To compensate for potential sample loss and to maintain statistical power, each group was supplemented with one additional animal (10%), resulting in a final sample size of four animals per group.

A total of 24 healthy male Wistar rats (Rattus norvegicus), aged 3 months and weighing 250 to 300 g, were used in this study. The rats were randomly allocated into six experimental groups ( *n* = 4 per group): control day—14, PMMA–HA day—14, SHED-seeded PMMA–HA scaffold day—14, control day—28, PMMA–HA day—28, and SHED-seeded PMMA–HA scaffold day—28. Randomization was performed using a simple lottery method to minimize selection bias. All rats were acclimatized for 1 week prior to intervention under standardized housing conditions (temperature: 21–23°C; humidity: 50 ± 5%; light/dark cycle: 12 hours). Each rat was housed individually in polycarbonate cages and provided with standard pellets and water *ad libitum* .

To induce alveolar bone defects, the maxillary anterior teeth of Wistar rats were extracted under general anesthesia using intramuscular ketamine (35 mg/kg; Kepro, the Netherlands) and xylazine (5 mg/kg; Xyla, the Netherlands). Following tooth extraction, treatment was administered according to group allocation. All surgical procedures were performed by three trained operators (T.S., R.D., F.W.P.) following a standardized protocol to ensure procedural consistency and minimize operator-dependent variability.

Following surgical procedures, all animals were monitored closely during the recovery period to ensure well-being and minimize discomfort. Postoperative analgesia was provided via subcutaneous meloxicam (1–2 mg/kg) once daily for up to 3 days, based on clinical indicators of pain or distress. Rats were housed individually in clean polycarbonate cages with soft bedding under standardized environmental conditions (temperature: 21–23°C; humidity: 50 ± 5%; 12-hour light/dark cycle). Daily assessments included food and water intake, grooming behavior, mobility, and general health status. Signs of infection, inflammation, or wound dehiscence were documented and managed as needed. Sutures were inspected regularly, with no adverse reactions observed during the healing period. Humane endpoints were defined in accordance with institutional guidelines. Euthanasia was performed on days 14 and 28 using an overdose of ketamine–xylazine, followed by cervical dislocation, in compliance with animal welfare standards (see Supplementary Materials , available in the online version only).

### Materials Preparation

HA powder was obtained from the Centre for Ceramics, Indonesia, where it is synthesized and extracted from natural limestone. PMMA granules were sourced from HiMedia Laboratories, India.

### Manufacture of PMMA–HA Scaffold

The PMMA–HA scaffold was prepared by combining 1 g of PMMA granules, 2 mL of acetone, and 4 g of HA powder to achieve a 20:80 weight ratio. PMMA was first dissolved in acetone and stored at –30°C for 24 hours to enhance polymer dispersion. Subsequently, HA powder was added to the PMMA solution and stirred using a magnetic stirrer until a homogeneous mixture was obtained. The composite was then cast into a Wistar rat tooth-shaped mold (26 mm ^3^ × 26 mm ^3^ × 3 mm ^3^ ) and subjected to freeze-drying. Final sterilization was performed using gamma irradiation at the Indonesian Nuclear Energy Agency (BATAN) (see Supplementary Materials , available in the online version only). 2

### Isolation and Culture of Stem Cells from Human Exfoliated Deciduous Teeth

SHED was isolated from a healthy, cavity-free, vital primary tooth with no root resorption and intact pulp, extracted for orthodontic reasons from an 8-year-old patient. The patient's identity was kept confidential, and parental consent was obtained. The pulp cavity was opened using a drill under aseptic conditions. The pulp tissue was then carefully preserved and transported to the Gedung Diagnostic Center Tissue Bank at Dr. Soetomo Hospital in Surabaya.

The cells were cultured in Dulbecco's Modified Eagle Medium (Life Technologies, Gibco BRL, United States), supplemented with 20% fetal bovine serum (Biochrom AG, Germany), 100 U/mL penicillin-G, 100 μg/mL streptomycin, 5 mL L-glutamine (Gibco Invitrogen, United States), and 100 μg/mL kanamycin (Gibco Invitrogen). The medium was changed after 3 days to eliminate unattached cells, and the remaining cells were transferred to a fresh medium containing fibroblast growth factor 2. Once the cells reached 80% confluence, they were detached using 0.05% trypsin-ethylenediaminetetraacetic acid (EDTA), washed, and recultured in 60- or 100-mm tissue culture dishes (Corning). These confluent cells were then prepared for research use. 2 The SHED used in this study had been previously characterized by Saskianti et al. 2

### Scaffold Application in Wistar Rats Socket

Following induction of alveolar bone defects in the maxillary socket using a standardized surgical protocol, PMMA–HA scaffolds seeded with SHED were carefully implanted into the defect site. Prior to transplantation, SHED at passage five were cultured in 24-well tissue culture plates. A 20-µL cell suspension containing 1 × 10 ^6^ cells was seeded into the PMMA-HA composite (80:20 ratio), based on previously reported *in vivo* dosing protocols. 2 The scaffold was gently inserted using sterile forceps, ensuring full contact with the surrounding bone margins and stable positioning within the socket. No additional fixation materials were used, as the scaffold's dimensions were tailored to fit snugly within the defects. Primary closure of the mucoperiosteal flap was achieved using interrupted 5.0 monofilament silk sutures (OneMed, Indonesia) to minimize tension and promote optimal healing. Postoperative care included administration of standard analgesics and daily monitoring for signs of infection or wound dehiscence. On postoperative days 14 and 28, animals were humanely euthanized in accordance with institutional ethical guidelines, and the maxillae were harvested for immunohistochemical analysis.

### Tissue Processing, Embedding, and Sectioning

Specimens were decalcified using a 10% EDTA solution (catalog no. 17892; Ajax Finechem, Thermo Fisher Scientific, Taren Point, Australia). Following decalcification, samples underwent overnight processing using a tissue processor (Leica TP1020, United States) and were subsequently embedded in molten paraffin wax (HistoCore Arcadia H Heated Paraffin Embedding Station, Leica). Serial sections were cut at a thickness of 5 µm using a rotary microtome (RM2235, Leica). Paraffin ribbons were flattened in a 40°C water bath and mounted onto polysine-coated microscope slides (Thermo Scientific), then dried at 60°C for 16 hours using a slide heater (Sakura, Tokyo, Japan). 20

### Immunohistochemistry Staining

Paraffin-embedded tissue sections (5 µm) were deparaffinized in xylene, rehydrated through graded ethanol, and subjected to antigen retrieval in citrate buffer (pH 6.0) using a pressure-heated chamber. Endogenous peroxidase activity was blocked with 0.5% H _2_ O _2_ in methanol for 20 minutes. Slides were placed in a moisture chamber and bordered with a PAP pen to contain reagents. Background Sniper was applied for 10 to 15 minutes.

Primary monoclonal antibodies (Universal Link) were applied at 1:500 dilution and incubated for 10 minutes, followed by PBS washing (2–5 minutes). Secondary antibody (Biogear) was added for 10 minutes, followed by another PBS wash. Trek Avidin-HRP Label was applied for 10 minutes, then washed again with PBS. Immunostaining was visualized using a DAB kit (cat. no. D7304-1SET, Sigma-Aldrich, United States), with substrate prepared by mixing 1 mL Betazoid buffer and one to two drops of DAB chromogen, incubated for 2 to 4 minutes. Slides were rinsed under running water (5–7 minutes).

Counterstaining was performed with Mayer's hematoxylin (2–3 minutes), followed by lithium carbonate (2–3 minutes) and a final water rinse (5–7 minutes). Sections were dehydrated in graded ethanol (80%, 96%, absolute), cleared in Xylene I–III (3 minutes each), and mounted with Entellan.

Antibodies used included BMP2 (1:500, cat. no. orb-10194), RUNX2 (1:500, cat. no. sc-390351), ALP (1:500, cat. no. sc-271431), TGF-β (1:500, cat. no. sc-130348), OCN (1:500, cat. no. GTX-13418), OPG (1:500, cat. no. sc-390518), RANKL (1:500, cat. no. sc-52950), and RANK (1:500, cat. no. LS-c368277). Expressions were assessed by counting the number of positive cells in five different fields at 400× magnification using a Nikon H600L light microscope (Japan). Location of the expression of markers was qualitatively assessed by two blinded, Indonesian-certified oral and maxillofacial pathologists (R.P.R., S.M.W.) to minimize observer bias. Interexaminer reliability was ensured through calibration sessions and consensus scoring conducted before analysis.

### Statistical Analysis

All statistical analyses were performed using IBM SPSS Statistics software, version 25.0 (IBM Corp., Armonk, New York, United States). Data normality was assessed using the Shapiro–Wilk test, and homogeneity of variance was evaluated using the Levene test. As the data were not normally distributed and showed unequal variances ( *p* < 0.05), non-parametric tests were applied. The Kruskal–Wallis test was used to compare differences among groups, followed by the Mann–Whitney *U* -test for pairwise comparisons. A *p* -value of < 0.05 was considered statistically significant for all markers analyzed, including BMP2, RUNX2, ALP, TGF-β, OCN, OPG, RANK, and RANKL.

## Results

The expressions of BMP2, RUNX2, ALP, TGF-β, OCN, OPG, RANK, and RANKL were evaluated on days 14 and 28 using a light inverted microscope at 400× magnification. This study shows that BMP2, RUNX2, ALP, TGF-β, OCN, and OPG were notably upregulated in the SHED-seeded PMMA–HA scaffold group compared to both the PMMA–HA and control groups. Conversely, RANK and RANKL were downregulated in the SHED-seeded PMMA–HA scaffold group relative to the other groups. Descriptive statistics and normality test results for marker expression are provided in Table 1 .

**Table 1:** Descriptive statistics and normality test results for marker expression (BMP2, TGF-β, RUNX2, RANK, RANKL, OPG, ALP, and OCN)

Group	BMP2	TGF-β	RUNX2	RANK	RANKL	OPG	ALP	OCN
K 14	4.50 ± 0.58	1.75 ± 0.50	1.50 ± 0.58	8.25 ± 0.96	8.75 ± 0.50	5.75 ± 0.96	3.75 ± 0.50	4.75 ± 0.96
Normality	0.024	0.001	0.024	0.272	0.001	0.272	0.001	0.272
K 28	5.00 ± 0.82	2.25 ± 0.50	2.00 ± 0.82	7.75 ± 0.96	8.25 ± 0.50	6.25 ± 0.50	4.25 ± 0.50	5.25 ± 0.96
Normality	0.683	0.001	0.683	0.272	0.001	0.001	0.001	0.272
P1 14	6.50 ± 1.73	3.00 ± 0.82	2.75 ± 0.50	6.75 ± 0.96	7.50 ± 0.58	7.75 ± 0.50	5.75 ± 0.96	6.25 ± 0.96
Normality	0.024	0.683	0.001	0.272	0.024	0.001	0.272	0.272
P1 28	7.00 ± 0.82	4.00 ± 0.82	3.75 ± 0.96	5.50 ± 0.58	6.25 ± 0.96	8.25 ± 0.96	6.25 ± 0.96	7.50 ± 0.58
Normality	0.683	0.683	0.272	0.024	0.272	0.272	0.272	0.024
P2 14	8.50 ± 0.58	5.50 ± 0.58	5.25 ± 0.96	3.00 ± 0.82	3.75 ± 1.50	9.00 ± 0.82	6.75 ± 0.96	8.00 ± 1.16
Normality	0.024	0.024	0.272	0.683	0.224	0.683	0.272	0.024
P2 28	8.75 ± 0.96	6.00 ± 0.82	5.75 ± 1.26	2.50 ± 0.58	3.25 ± 0.50	9.50 ± 0.58	6.75 ± 0.96	8.50 ± 1.00
Normality	0.272	0.683	0.406	0.024	0.001	0.024	0.272	0.001

Abbreviations: ALP, alkaline phosphatase; BMP2, bone morphogenetic protein 2; OCN, osteocalcin; OPG, osteoprotegerin; RANK, receptor activator of nuclear factor kappa-Β; RANKL, receptor activator of nuclear factor kappa-Β ligand; RUNX2, runt-related transcription factor 2; TGF-β, transforming growth factor beta.

Note: Values expressed as mean ± standard deviation. Normality assessed using the Shapiro-Wilk test.

On day 14, BMP2, TGF-β, and RUNX2 showed increased expression in the SHED-seeded PMMA–HA scaffold group compared to the PMMA–HA group ( Fig. 1 ). RANK and RANKL expression levels were already reduced at this time point, consistent with the study hypothesis ( Fig. 2 ). Additionally, OPG, OCN, and ALP were elevated in the SHED-seeded PMMA–HA scaffold group compared to the control group on day 14 ( Fig. 3 ).

**Fig. 1:**
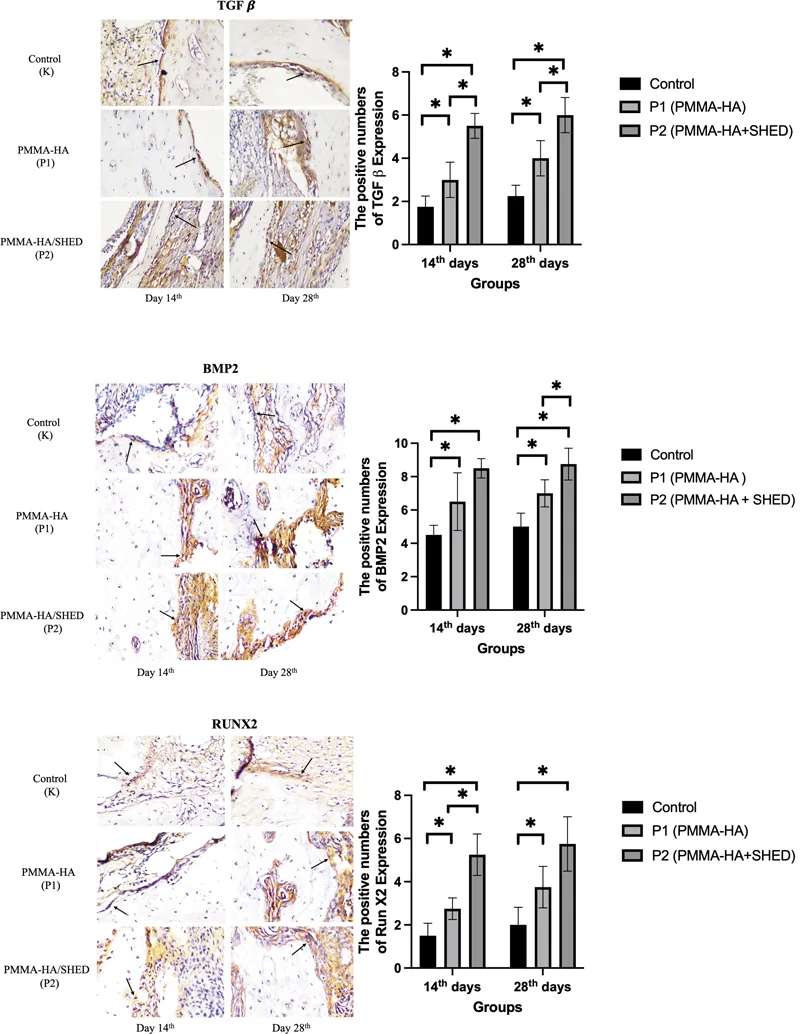
Immunoreactivity of BMP2, TGF-β, and RUNX2 expression was upregulated (intense brown staining) in the osteoblast cells (arrow) in PMMA–HA (P1) and PMMA–HA/SHED (P2) groups compared with the control (K) (* *p* < 0.05).

**Fig. 2:**
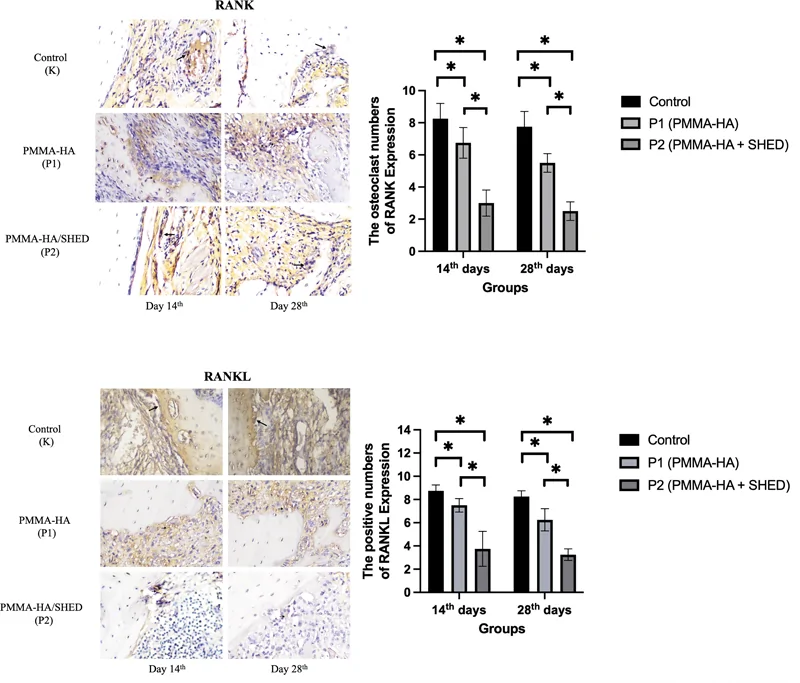
Immunoreactivity of RANK and RANKL expression was downregulated (intense brown staining) in the osteoclast cells (arrow) in PMMA–HA (P1) and PMMA–HA/SHED (P2) groups compared with the control (K) (* *p* < 0.05).

**Fig. 3:**
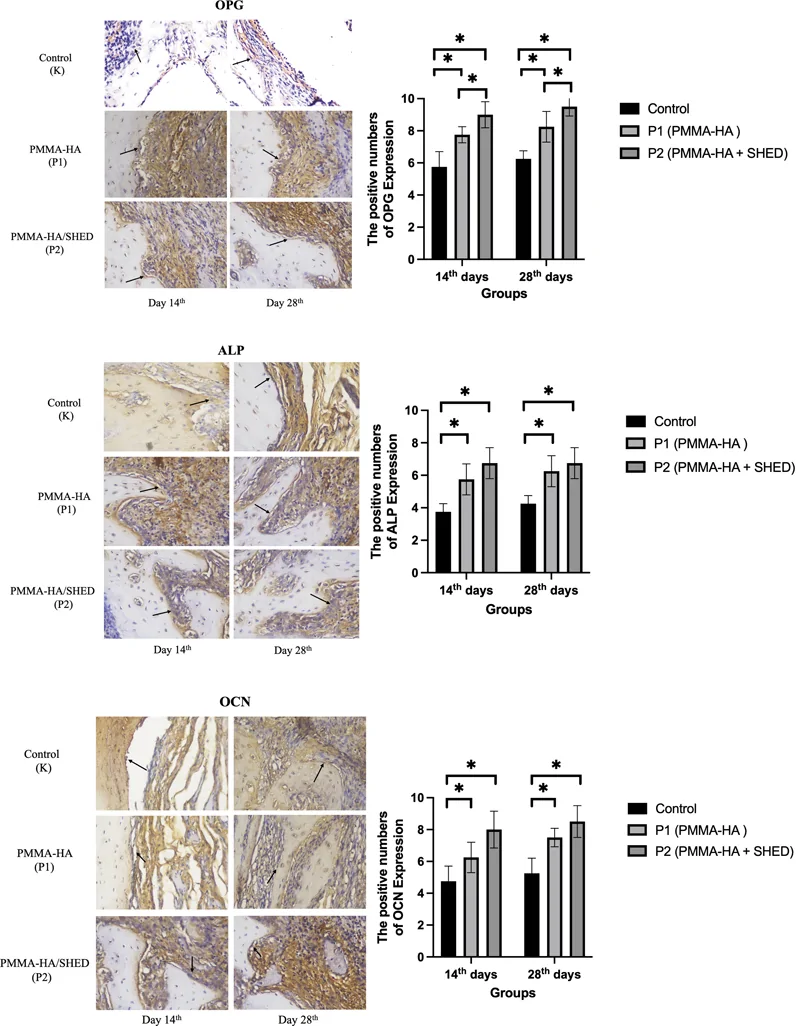
Immunoreactivity of OPG, ALP, and OCN expression was upregulated (intense brown staining) in the osteoblast cells (arrow) in PMMA–HA (P1) and PMMA–HA/SHED (P2) groups compared with the control (K) (* *p* < 0.05).

## Discussion

Alveolar bone defects commonly result from dental trauma, congenital defects, and periodontal disease. If left untreated, these defects may lead to progressive loss of alveolar bone support and compromise the structural integrity and function of the oral cavity. 1 A range of treatment techniques has been proposed, including bone grafts, directed tissue regeneration, and a combination of these procedures. Alveolar bone graft treatment is essential for restoring the shape, function, and structural integrity of the alveolar bone. 21 Despite various approaches, unequivocal success has not yet been achieved. Autogenous bone transplantation surgery may cause substantial trauma and potentially affect the function of the patient's original tissue structure. Hence, novel technologies based on tissue engineering (using stem cells and scaffolding) may emerge as possible therapies. 1

The current study demonstrated the feasibility of SHED seeded in PMMA–HA scaffold for tissue engineering by promoting alveolar bone defect repair, possibly through the stimulation of bone formation. Immunohistochemical analysis revealed an upregulation of BMP2, RUNX2, ALP, TGF-β, OCN, and OPG—proteins that regulate osteoblastic differentiation—and a simultaneous downregulation of osteoclastic proteins in the RANK/RANKL axis in the SHED-seeded PMMA–HA scaffold group compared with the control and PMMA–HA-only groups on the 14th and 28th days.

SHEDs in the current study are a multipotent MSC population from exfoliated primary teeth, which highly expressed MSC markers (CD90 and CD105) and lacked hematopoietic markers (CD45 and CD73) expression. 3 Moreover, the presence of calcium deposits as detected by Alizarin Red staining validated the osteogenic potential of SHED, which has been shown to be supported by composite scaffolds such as PMMA–HA. 2

MSCs contribute to bone repair by modulating key signaling pathways, notably the BMP/TGF-β axis. BMP2, a member of the TGF-β superfamily, plays a pivotal role in initiating osteoblast differentiation. 17 22 In this study, the SHED–PMMA–HA group exhibited significantly elevated BMP2 expression on day 28 and increased TGF-β levels on both days 14 and 28, compared to the PMMA–HA only group. This upregulation was accompanied by a marked increase in RUNX2 expression on day 14, a transcription factor downstream of BMP2 and TGF-β that is essential for osteoblastogenesis. The combination of SHED and PMMA–HA scaffold clearly initiated bone regeneration as early as day 14, with sustained signaling activity through day 28. These findings are consistent with the previous study by Saskianti et al, which showed enhanced BMP2 and TGF-β expression in SHED–carbonate apatite constructs compared to scaffold-only controls. 8

The presence of RUNX2 is essential for osteoblast maturation by regulating ALP and OCN. 22 23 In this study, ALP and OCN expression levels were markedly elevated in the SHED–PMMA–HA group compared to the control group, indicating active bone formation mediated by mature osteoblasts. 8 22 These findings are consistent with previous reports, including those by Saskianti et al, which demonstrated significantly higher OCN expression in the SHED–carbonate apatite group relative to the CAS-only group. 8 The upregulation of ALP and OCN further supports the hypothesis that SHED enhances osteoconduction by promoting early osteoblastic differentiation.

SHED has been shown to promote osteogenic differentiation, particularly enhancing osteoblast activity in alveolar bone defects. 24 25 Concurrently, PMMA–HA scaffolds support osteoblast proliferation due to their favorable porosity and surface roughness. 26 Pore sizes ranging from 100 to 300 µm are considered optimal for initiating bone formation, facilitating osteochondral development, and promoting cell attachment. 8 27 28 29 The presence of phosphate ions (PO _4_^3−^ ) contributes to tissue growth and suppresses osteoclast activity, while hydroxyl groups (OH ^−^ ) enhance ionic conduction and accelerate cytoskeletal remodeling in osteoblasts. 30 Moreover, SHED-seeded PMMA–HA may also have played a role in the present study by promoting wound healing from recruitment of fibroblasts, lymphocytes, and plasma cells in addition to pro-osteogenic and anti-osteoclastic effects. 5 24

In the present study, OPG expression was significantly increased in the PMMA–HA scaffold seeded with SHED at days 14 and 28, compared to the PMMA–HA scaffold alone. In contrast, the expression levels of RANK and RANKL were significantly decreased in the SHED-seeded group at the same time point. These findings support the hypothesis that SHED, when integrated into a PMMA–HA scaffold, can modulate bone remodeling by enhancing OPG activity while downregulating RANK and RANKL. The OPG/RANK/RANKL triad plays a fundamental role in regulating the balance between osteoblast and osteoclast activity. OPG, produced by osteoblasts, functions as a soluble decoy receptor that binds to RANKL, thereby preventing its interaction with RANK on pre-osteoclasts. This blockade inhibits osteoclastogenesis and favors bone formation. 31 32 Several studies have shown the opposing effects of RANKL and OPG on bone turnover. 8 15

The observed downregulation of RANK/RANKL in this study could be mediated by SHED secreted proangiogenic factors and interleukins with pro- or anti-inflammatory effects. 8 33 34 35 These findings suggest that SHED may contribute to bone regeneration by modulating the local inflammatory microenvironment during biomaterial implantation. 33 34 However, further research on cytokine and inflammatory marker analysis is needed. SHED thus represents a promising cell source in tissue engineering, capable of enhancing bone regeneration through recruitment of host osteoprogenitor cells and differentiation into osteoblasts. 5 8

Previous studies by Saskianti et al 30 and Komang-Agung et al 36 have demonstrated that elemental components of PMMA–HA scaffolds—such as C, O, Al, Si, P, Na, Mg, and Ca—play a key role in promoting osteoblast proliferation and extracellular matrix formation, while concurrently inhibiting osteoclast activity and bone resorption. 30 36 These bioactive properties support cell viability and enhance biological processes essential for bone regeneration, positioning PMMA–HA as a promising synthetic graft material in regenerative dentistry.

The limitation of this study is that it was an observational immunohistochemical analysis of osteogenic and osteoclastic marker expression using a limited sample size and time frame. Future research should incorporate larger cohorts, extended time frames, and morphometric assessments of new bone formation to better elucidate the regenerative potential of SHED–PMMA–HA scaffolds. Additionally, molecular techniques such as polymerase chain reaction and enzyme-linked immunosorbent assay are recommended to quantify gene and protein expression, thereby clarifying the mechanisms underlying SHED-mediated osteogenesis.

## Conclusion

SHED and PMMA–HA scaffolds demonstrate promising potential in regenerative medicine by enhancing osteogenesis in induced alveolar bone defect animal models. The observed upregulation of BMP2, RUNX2, ALP, TGF-β, OCN, and OPG, along with the downregulation of RANK and RANKL, supports their role in promoting bone formation and reducing resorption. Given that SHED is derived from exfoliated deciduous teeth and possesses high proliferative and immunomodulatory capacity, these findings may hold particular relevance for dental applications, where minimally invasive and biologically compatible therapies are especially valuable.
