# Supplementary material for: Osteogenic Marker Expression after SHED and PMMA–HA Scaffold Application in Alveolar Bone Defects
Source: Eur J Dent. 2025 Nov 17;20(4):1143–51. doi: 10.1055/s-0045-1812311 (PMC13623490; doi:10.1055/s-0045-1812311)
Supplement: Supplementary file 1 [file 10-1055-s-0045-1812311-s2574374.pdf]

1. Stem cell from human exfoliated deciduous teeth isolation protocol.

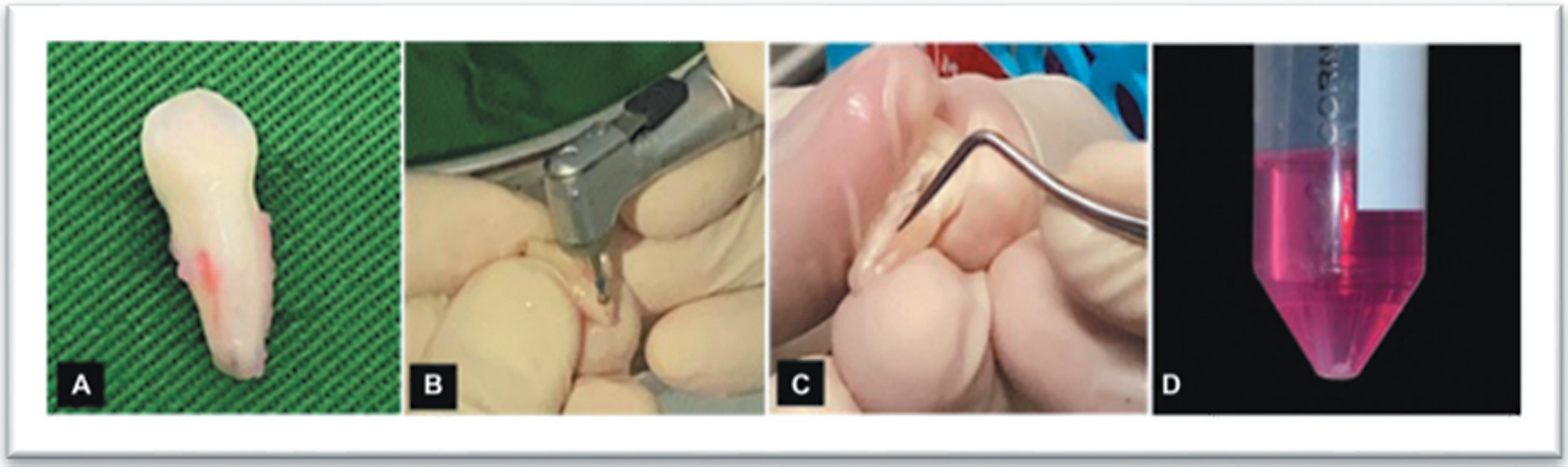

(**A**) #73 persistence of deciduous tooth, free of cavities, and no root resorption. (**B–C**) The dental pulp cavity was opened using drills with a sterilized round bur. (**D**) Pulp tissue was taken in the medium within a 15-mL conical tube.

2. Mixed polymethylmethacrylate and hydroxyapatite material.

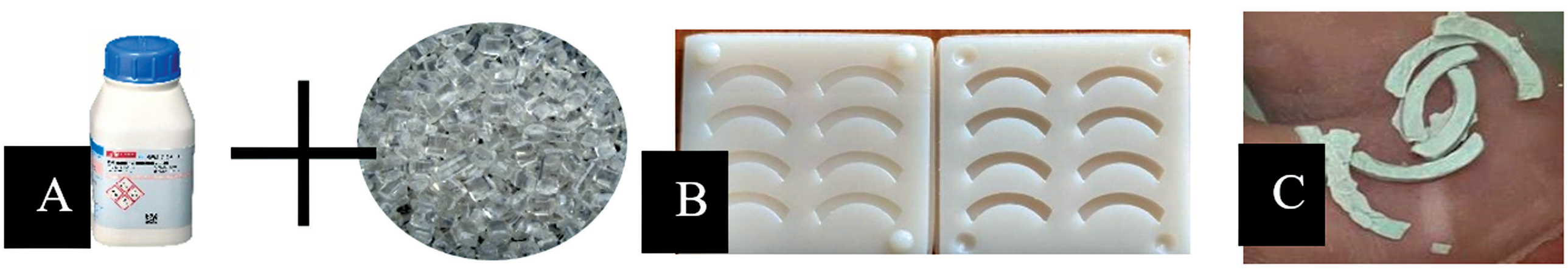

(**A**) Hydroxyapatite from Balai Besar Keramik, Indonesia + polymethylmethacrylate granules. (**B**) Wistar rat tooth-shaped mold. (**C**) The final shape of PMMA-HA scaffold.

 © 2025. The Author(s).

3. Animal experimental preparation and SHED and PMMA-HA scaffold application.

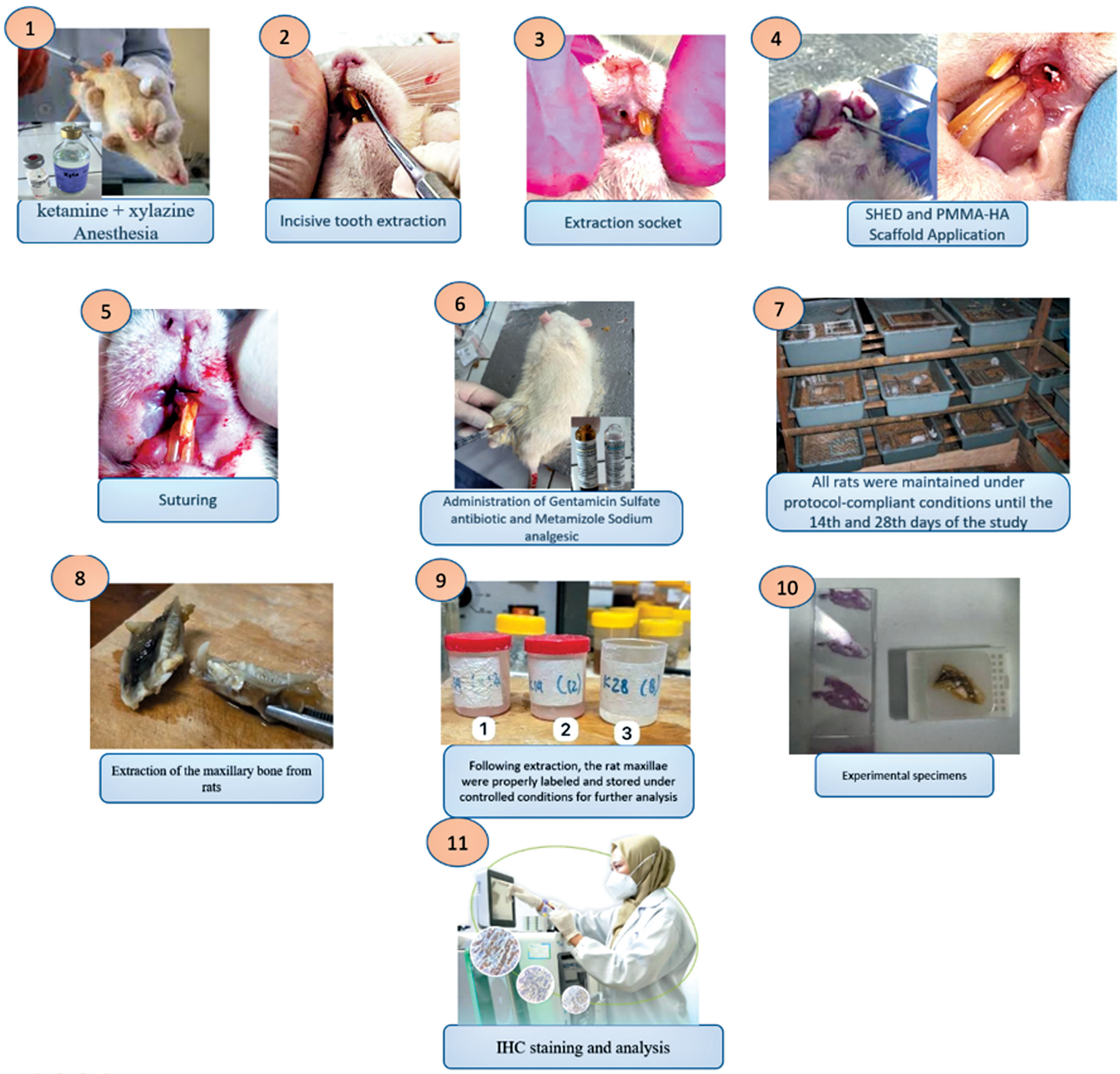

 © 2025. The Author(s).
